# Harmonizing qualitative and quantitative analyses in scRNA-seq via information geometry

**DOI:** 10.1093/bib/bbaf631.055

**Published:** 2025-12-12

**Authors:** Jinpu Cai, Yuxuan Wang, Yunhao Qiao, Cheng Wang, Ziqi Rong, Luting Zhou, Haoyang Liu, Jingyi Jessica Li, Hongyi Xin

**Affiliations:** Global College, Shanghai Jiao Tong University, Shanghai, China; Global Institute of Future Technology, Shanghai Jiao Tong University, Shanghai, China; Global Institute of Future Technology, Shanghai Jiao Tong University, Shanghai, China; Global Institute of Future Technology, Shanghai Jiao Tong University, Shanghai, China; Bioinformatics Interdepartmental Program, University of California, Los Angeles, CA, United States; Global College, Shanghai Jiao Tong University, Shanghai, China; Global Institute of Future Technology, Shanghai Jiao Tong University, Shanghai, China; Biostatistics Program, Public Health Sciences Division, Fred Hutchinson Cancer Center, Seattle, WA, United States; Department of Statistics and Data Science, University of California, Los Angeles, CA, United States; Global Institute of Future Technology, Shanghai Jiao Tong University, Shanghai, China

## Abstract

Single-cell and spatial transcriptomics enable high-resolution characterization of cellular states, but standard analyses often rely on Euclidean or log-transformed distances that distort cell-to-cell relationships. Euclidean distances on normalized counts overemphasize highly expressed genes, while log transformations amplify qualitative on/off differences and are sensitive to sequencing depth.

To overcome these limitations, we introduce GAIA (Geometric Analysis from an Information Aspect), an information-geometric framework that models each cell as a multinomial distribution over genes. Distances between cells are measured using the Fisher-Rao metric, which reduces to angular distances on a unit hypersphere after a square-root transformation. This geodesic approach provides a principled, interpretable, and computationally efficient similarity measure.

GAIA naturally reconciles qualitative and quantitative gene expression variation: subtle quantitative changes correspond to smooth displacements along the manifold, while qualitative transitions induce larger geodesic separations. It preserves robust and consistent cell-to-cell relationships, mitigates sequencing-depth effects and reduces the need for labor-intensive gene selection. In spatial transcriptomics, GAIA amplifies nuanced transcriptomic differences between spots, improving domain segmentation. Overall, GAIA offers a knowledge-lean, variance-stabilizing framework for analyzing single-cell and spatial transcriptomic data, enhancing the resolution of cell type and state identification.

